# Osteopontin Fragments with Intact Thrombin-Sensitive Site Circulate in Cervical Cancer Patients

**DOI:** 10.1371/journal.pone.0160412

**Published:** 2016-08-05

**Authors:** Danny T. M. Leung, Pak-Leong Lim, Tak-Hong Cheung, Raymond R. Y. Wong, So-Fan Yim, Margaret H. L. Ng, Frankie C. H. Tam, Tony K. H. Chung, Yick-Fu Wong

**Affiliations:** 1 Clinical Immunology Unit, The Chinese University of Hong Kong, Hong Kong SAR, China; 2 IgGENE, Fo Tan, Hong Kong SAR, China; 3 Department of Obstetrics and Gynaecology, The Chinese University of Hong Kong, Hong Kong SAR, China; 4 Department of Obstetrics, Gynaecology and Reproductive Biology, Brigham and Women’s Hospital, Harvard Medical School, Boston, MA, United States of America; 5 Department of Anatomical and Cellular Pathology, The Chinese University of Hong Kong, Hong Kong SAR, China; University of Alabama at Birmingham, UNITED STATES

## Abstract

We investigated whether circulating osteopontin (OPN) could be used as a biomarker for cervical cancer. We employed a monoclonal antibody (mAb 659) specific for the unique and intact thrombin-sensitive site in OPN using an inhibition ELISA. We found significantly higher levels of OPN in 33 cervical cancer patients in both the plasma (mean +/- SD, 612 +/- 106 ng/mL) and serum (424 +/- 121 ng/mL) compared to healthy subjects [409 +/- 56 ng/mL, from 31 plasma samples (*P* < 0.0001), and 314 +/- 98 ng/mL, from 32 serum samples (*P* = 0.0002), respectively]. Similar results were obtained when the plasma from a bigger group (147 individuals) of cervical cancer patients (560 +/- 211 ng/mL) were compared with the same plasma samples of the healthy individuals (*P* = 0.0014). More significantly, the OPN level was highest in stage III-IV disease (614 +/- 210 ng/mL, from 52 individuals; *P* = 0.0001) and least and non-discriminatory in stage I (473 +/- 110 ng/mL, from 40 individuals; *P* = 0.5318). No such discrimination was found when a mAb of a different specificity (mAb 446) was used in a similar inhibition ELISA to compare the two groups in the first study; a commercial capture ELISA also failed. The possibility that the target epitope recognized by the antibody probe in these assays was absent from the circulating OPN due to protein truncation was supported by gel fractionation of the OPN found in patients’ plasma: 60–64 kDa fragments were found instead of the presumably full-length OPN (68 kDa) seen in healthy people. How these fragments are generated and what possible role they play in cancer biology remain interesting questions.

## Introduction

Osteopontin (OPN), known previously as SPP1 (secreted phosphoprotein 1) or ETA-1 (early T-lymphocyte activation protein 1), was initially identified as an extracellular matrix protein produced by osteoclasts [1]. It is now considered to be a pleiotropic, pro-inflammatory cytokine produced by a wide variety of cells including epithelial cells, endothelial cells, kidney cells, B and T cells, NK cells, Kupffer cells, dendritic cells and macrophages [2]. As a member of the family of small integrin-binding ligand, N-linked glycophosphoproteins (SIBLINGs), OPN is highly conserved among mammals [3].

Human OPN has 314 amino acids, including a unique and conserved thrombin-sensitive site. Cleavage of this site by thrombin, which normally occurs in the circulation, fragments the protein roughly into two equal halves, each with different biological activities. The amino-terminal half of the protein binds to a variety of cell surface integrins, such as αvβ1, αvβ3, αvβ5, αvβ6, and α8β1, through the arginine-glycine-aspartate (^159^RGD^161^) motif in the protein, and also with α4β1 and α9β1 integrins, through the thrombin-generated site (^162^SVVYGLR^168^). The carboxy-terminal half interacts with the CD44 cell surface splice variants, CD44v6 and CD44v3 [4, 5]. Through these various extracellular interactions, thrombin-activated OPN plays pivotal roles in diverse physiological processes, such as bone remodeling, inflammation, and wound healing [6], as well as in various pathologies, including autoimmunity [7–9] and tumor metastasis [10] or progression [11, 12]. Thrombin-activated OPN has also been reported to inhibit the apoptosis in, or promote the survival and proliferation of, cancer cells [3].

More recently, OPN was shown to exert an intracellular function which can affect diverse cellular processes such as tumor progression [13] and interferon-α production in dendritic cells [14]. Here, too, these functions require the OPN protein to be appropriately cleaved, not by thrombin, but by various caspases [15] or produced as appropriate truncates from RNA splice variants [16–18]. Extracellularly, various metalloproteinases (MMPs) also cleave OPN into various types of fragments which have various tumorigenic or biological activities [19, 20].

Overexpression of OPN in the form of mRNA transcripts or intracellular proteins was observed in tumor tissues derived from breast or lung cancer [21, 22], ovarian cancer [23], and cervical cancer [24–27]. Significantly increased levels of circulating OPN were also reported for several types of cancer including cervical cancer [26], prostate cancer [28], and colorectal cancer [29]. However, there were conflicting reports regarding head and neck squamous cell carcinoma [30, 31]. In addition, different diagnostic kits were found to yield quite different results from one another for the same patient samples [32]. We were interested to use this non-invasive method to examine our cervical cancer patients. This type of cancer is very common in Hong Kong [24]; it is highly invasive and lethal since the disease can progress rapidly and asymptomatically from preclinical-lesion to overt cancer. We were disappointed with the results obtained initially using a commercial OPN detection kit, and consequently decided to devise our own detection assays. We herein describe the development of such an assay based on the use of a unique monoclonal antibody and an assay format that is used infrequently–inhibition ELISA.

## Materials and Methods

### Cells and Cell Culture

Sf9 cell line derived from the fall armyworm *Spodoptera frugiperda* was obtained from Invitrogen and maintained in Grace’s insect cell medium supplemented with 10% FCS, antibiotics (100 IU/mL penicillin, 100 μg/mL streptomycin) and 2.5 μg/mL amphotericin B at 27°C. The human promyelocytic leukemia cell line HL60 and murine plasmacytoma NS0 were obtained from American Type Culture Collection and maintained in RPMI 1640 medium containing 10% FCS, 100 IU/mL penicillin and 100 μg/mL streptomycin at 37°C and 5% CO_2_.

### Antibodies and Reagents

HRP-coupled goat anti-mouse immunoglobulin (H + L) and HRP-coupled streptavidin were from Zymed (San Francisco, CA). NS0-derived recombinant human full-length OPN were purchased from R & D Systems (Minneapolis, MN) (Cat. No.: 1433-OP) and from Sigma (St. Louis, MD) (Cat. No.: O4264). Thrombin and glutathione-coupled sepharose 4B beads were obtained from Amersham Biosciences (Piscataway, NJ). Protein G-coupled sepharose 4B beads, protease inhibitor cocktail, PMSF, TMB, PMA, FITC-conjugated goat anti-mouse Ig antibodies, and N-hydroxy-sucinimidobiotin were obtained from Sigma. Bio-Gels of various retention limit [P10 (20 kDa), P30 (40 kDa), P60 (60 kDa), P100 (100 kDa)] were obtained from Bio-Rad Laboratories (Berkeley, CA). Human OPN Assay Kit was obtained from Immuno-Biological Laboratories (IBL) Co. Ltd., Gunma, Japan.

### Patients and Specimens

Pre-operative paired plasma and serum samples were obtained with consent from a first cohort of 33 patients diagnosed with cervical cancer [27 patients with squamous cell carcinoma, 6 patients with adenocarcinoma; ages ranging from 27–82 years (mean +/- SD = 52.3 +/- 12.4 years)], and from a second cohort of 147 cervical cancer patients [40 patients with disease stage I, ages ranging from 39–80 years (mean +/- SD = 57.7 +/- 12.2 years); 55 patients with disease stage II, ages ranging from 31–90 years (mean +/- SD = 62.5 +/- 15.5 years); 52 patients with disease stage III-IV, ages ranging from 35–92 years (mean +/- SD = 64.5 +/- 15.0 years)], from the Department of Obstetrics and Gynecology, Prince of Wales Hospital, Hong Kong. As controls, 31 plasma samples from healthy women (ages ranging from 19–92 years (mean +/- SD = 55.1 +/- 21.6 years) and 32 serum samples (ages ranging from 14–86 years (mean +/- SD = 42.0 +/- 16.9 years) sent for routine laboratory test were used. All samples were stored at -80°C before use. All the data were analyzed anonymously. This study was approved by the The Joint Chinese University of Hong Kong—New Territories East Cluster Clinical Research Ethics Committee.

### Bacterial or Insect-Cell Derived Recombinant Human OPN

Bacterially derived recombinant human OPN of N-terminal and C-terminal half were generated as GST fusion proteins, OPN-O-GST and OPN-P-GST, respectively, in *Escherichia coli* BL21 using the pGEX-2T expression vector (Amersham Biosciences) as previously described [33]. cDNA obtained from HEp-2 cells was PCR-amplified to generate DNA fragments using the following forward (F) primers and reverse (R) primers: OPN-O [(aa 1–175), F, 5’- CGTGGATCCATGAGAATTGCAGTGATTTGC—3’(containing a BamH1 site, underlined); R, 5’- CGATGAATTCCGCGAAACTTCTTAGATTTTGA– 3’ (containing an EcoR1 site, underlined)], and OPN-P [(aa 169–314), F, 5’- CGTGGATCCTCAAAATCTAAGAAGTTTCGC– 3’ (containing a BamH1 site, underlined); R, 5’- AATTCCCGGGGATTAATTGACCTCAGAAGATGC– 3’ (containing a SmaI site, underlined)] and subcloned into the pGEX-2T vector. The fusion proteins derived from the above constructs were induced with isopropyl-β-D-thiogalactopyranoside, purified by affinity chromatography, and examined on SDS-PAGE by Commassie Blue staining and WB.

Insect-cell derived recombinant human OPN were generated as V5 peptide-tagged (V5) fusion proteins (OPN-O-V5 or OPN-P-V5) in Sf9 using the BaculoDirect Baculovirus Expression System according to manufacturer’s instruction (Invitrogen). cDNA obtained from HEp-2 cells was PCR-amplified to generate DNA fragments using the following forward (F) primers (containing a Kozak sequence, underlined) and reverse (R) primers: OPN-O (F, 5’- CCACCATGGGAATGAGAATTGCAGTGATTTGC– 3’; R, 5’- GCGAAACTTCTTAGATTTTGA– 3’), and OPN-P (F, 5’- CCACCATGGGATCAAAATCTAAGAAGTTTCGC– 3’; R, 5’- ATTGACCTCAGAAGATGCACT– 3’) and subcloned into the entry vector pCR8/GW/TOPO (Invitrogen). Purified plasmid with insert of right orientation was recombined with the BaculoDirect linear DNA by incubation with LR clonase overnight at room temperature. Sf9 cells growing at log-phase were transfected with the recombined DNA mixture by Cellfectin (Invitrogen) and selected with 100 mM ganciclovir. Low titer of virus preparation (P1 viral stock) bearing the OPN-O or OPN-P gene constructs were harvested from the spent culture supernatant at day 14–21 post-transfection under ganciclovir selection, where less than 10% of Sf9 cells was infected as revealed by anti-V5 antibody staining in IFA. High titer virus preparation (P2 viral stock) was harvested from Sf9 cells inoculated with an aliquot of P1 viral stock for 5–7 days under ganciclovir selection where 50–80% of cells were infected. Fusion proteins were obtained from lysates of Sf9 cells incubated with P2 viral stock without selection (72 hr at 27°C) where almost 100% of cells were infected at the time of harvest as revealed by anti-V5 antibody staining. Cytosolic extracts were prepared by incubating the infected Sf9 cells with lysis buffer (1% NP-40, 150 mM NaCl, 10 mM Tris, pH 7.5, 1X protease inhibitor, 1 mM PMSF) at 4°C for 30 min, followed by centrifugation (14,000 rpm, 10 min, 4°C)

### HL60-Derived OPN

The culture supernatant of HL60 (5 x 10^6^/mL) cells with or without treatment with PMA (ng/mL) for 72 hr was harvested and concentrated 1:10 the original volume by centrifugation at 13,000 rpm for 20 min at 4°C (Centricon YM-10, 10 kDa cutoff; Millipore, Billerica, MA). Samples were stored at -80°C before analysis.

### OPN-Specific Monoclonal Antibodies (mAbs)

BALB/c mice were hyperimmunized with purified bacterially-derived OPN-O-GST or OPN-P-GST protein, and spleen cells obtained from these animals were fused with NS0 myeloma cells as described [34]. Hybridomas obtained were screened for reactivity against insect cell-derived recombinant proteins (OPN-O-V5 or OPN-P-V5) by direct-binding ELISA; positive hybridomas were further characterized by IFA and WB. Monoclonal antibodies from selected clones (mAb 659, mAb 446, mAb 492) were obtained from the spent-culture supernatant or ascites fluid and purified by ammonia sulfate precipitation. In some cases, the antibodies were protein-G selected and biotinylated using N-hydroxy-sucinimidobiotin.

## ELISA

### I. Inhibition ELISA

OPN was detected by determining the ability of the test sample (human serum or plasma, or HL60-derived culture supernatant) to block the binding of the OPN-specific indicator antibody (mAb 659, mAb 446 or mAb 492) to the insolubilized antigen [bacterially-derived OPN-O-GST (for mAbs 659 and 446) or OPN-P-GST (for mAb 492); NS0-derived full-length OPN]. The concentration of indicator antibody used was determined previously by titration of the antibody against the respective antigen, based on 70% OD_max_. Thus, wells of 96-well ELISA plates (Immunlon II; Dynatech, Chantilly, VA) were coated with the different antigens (1 μg/mL) at 4°C using bicarbonate buffer (pH 9.6). Fifty μL of diluted sample (for plasma and serum, dilution ratio = 1:16.6) were mixed with 50 μL of diluted indicator antibody, and incubated with the immobilized antigen for 16 hr at 4°C. After washing, bound indicator antibody was detected using HRP-conjugated goat anti-mouse Ig (1:1000) (1.5 hr at 37°C) and TMB, and the results read at OD 450 nm in an ELISA reader. A calibration curve was constructed for each experiment using 8 serial dilutions of full-length OPN (5 to 625 ng/mL).

### II. Competitive ELISA

This was employed to determine whether mAb 659 and mAb 446 shared a common binding site in OPN. Thus, 50 μL of serially diluted mAb 659 (competitor) were mixed with 50 μL of biotin-coupled mAb 446 of a pre-determined concentration and incubated with immobilized OPN-O-GST at 4°C overnight. Cold (unbiotinylated) mAb 446 was used as positive control. Following washing, bound indicator antibody was detected by incubation with 100 μL HRP-conjugated streptavidin (1:1000) for 1.5 hr at 37°C, and the reaction developed using TMB; the results were read at OD 450 nm. Binding specificity was further confirmed by a reverse assay, using mAb 446 as competitor, and biotin-coupled mAb 659 as indicator.

### Indirect Immunofluorescence Assay (IFA)

Cytospin preparations of Sf9 cells expressing OPN-O- or OPN-P-V5 protein were methanol-fixed, blocked with 2.5% BSA and stained with the reagent mAb for 30 min at room temperature. Following incubation with FITC-conjugated goat anti-mouse Ig antibodies (1:40) for 30 min at room temperature, the cells were examined by UV microscopy [35]. Anti-V5 antibody (Invitrogen) was used as a positive control.

### Western Blotting (WB)

Recombinant proteins or Sf9 cell lysates were treated in reducing sample buffer at 95°C for 5 min, resolved on 10–12.5% SDS-PAGE gel, and then transferred to PVDF membranes. The membranes were blocked with 2.5% BSA and later incubated with the detecting mAb (mAb 659 or mAb 446) at 4°C overnight; following this, the membrane was incubated with HRP-conjugated goat anti-mouse Ig antibodies for 2 hr at room temperature. Membranes were then thoroughly washed and used for film development by ECL chemoluminescence (Amersham Biosciences).

### Thrombin Digestion Assay

Full-length OPN (25 ng) was incubated with graded doses of thrombin (5, 2.5, 1.25, 0.63, 0.31, and 0.16 U/mL) in Tris-buffer, pH 8.0 at 37°C for 1 hr. After heating with reducing sample buffer at 95°C for 5 min, the samples were resolved on 10–12.5% SDS-PAGE gel, transferred to PVDF membranes, and examined by WB. In the mAb-protection study, OPN (25 ng) was pre-incubated with graded doses of mAb 659 in Tris-buffer, pH 8.0, at 4°C overnight, followed by incubation with 5 U/ml thrombin at 37°C for 1 hr. Samples were then treated with reducing sample buffer at 95°C for 5 min, resolved on 10–12.5% SDS-PAGE gel, transferred to PVDF membranes, and examined by WB.

### Gel Chromatography

Mini-columns (1 mL bed-size) were prepared using Bio-Gel (Bio-Rad Laboratories, Berkeley, CA) of different retention limits: P10 (20 kDa), P30 (40 kDa), P60 (60 kDa), P100 (100 kDa). These were thoroughly equilibrated with PBS (100 μl) by repeated (5X) loading and subsequent centrifugation (200 x *g*, 2 min). Each column was first calibrated using blue dextran and DNP-lysine. In the experiment, 100 μL human plasma was loaded to each column and centrifuged (200 x *g*, 2 min). The eluate (100 μL) was collected and the column was loaded with PBS (100 μL) again, centrifuged and the eluate collected. The procedure of PBS-loading was repeated, so that, for each sample, 10 such eluates (fractions) were obtained. The OPN concentration in each fraction was determined using the mAb 659 inhibition ELISA. As a control, bovine serum albumin (2 mg/mL) was also run through each column using the same procedure, but the protein content in the fractions was determined using the bicinchoninic acid (BCA) protein assay kit (Pierce, Rockford).

### OPN Gene Expression Assay

Microdissection of the tumor tissue, RNA extraction from the processed tissue, as well as reverse transcription and real-time PCR of the extracted RNA, were all performed as described [24].

### Statistics

Comparison of difference between two groups was performed using the unpaired *t*-test. Correlation between groups of data was performed using regression analysis, and a *p*-value of <0.05 was considered statistically significant. Variable data were expressed by scatterplot analysis (GraphPad Prism 3, GraphPad Software, Inc., San Diego, CA).

## Results

### Monoclonal Antibodies Are Specific for Different Sites in OPN

**Fig 1:**
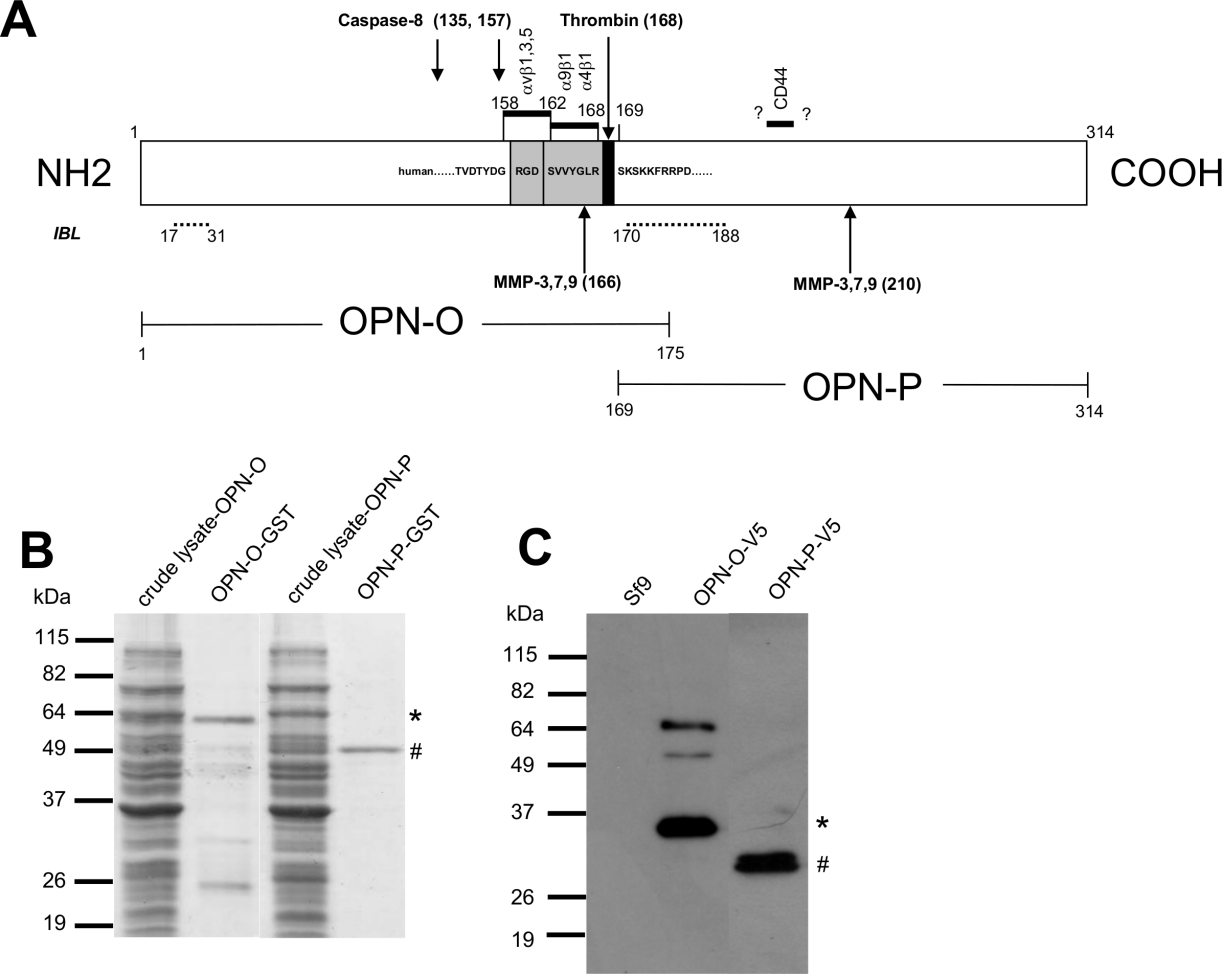
Types of OPN antigens and their characterization. A) Map of human OPN protein showing the various fragments (OPN-O, OPN-P) produced as GST fusion proteins in bacteria and as V5-tagged fusion proteins in insect cells. Numericals denote amino-acid numbering of protein. Shown are the cleavage sites of caspase-8 and various metalloproteinases (MMP-3, -7, -9), the integrin binding sites (αvβ1, αvβ3, αvβ5, α9β1 and α4β1) and the CD44-binding site. Binding sites of the pair of antibodies used in the OPN detection kit marketed by IBL, Gunma, Japan, are indicated by dashed lines. The sequence surrounding the thrombin-sensitive site is also shown. B) Demonstration of the purity of the bacterially-derived recombinant fusion proteins (OPN-O-GST and OPN-P-GST) isolated by affinity chromatography, separated on 12.5% SDS-PAGE gel and stained by Coomassie Blue. The starting material (crude lysate), the OPN-O-GST antigen (*), the OPN-P-GST antigen (#), and mol. wt. markers, are shown. C) WB results identifying the fusion proteins (OPN-O-V5 [*], OPN-P-V5 [#]) derived from insect cells. Cell lysates were separated on 12.5% SDS-PAGE gel and probed with anti-V5 antibody. Lysate of un-infected insect cells (Sf9) included as negative control.

Recombinant OPN proteins were produced in *E*. *coli* from DNA cloned from HEp-2 cells. Thus, two GST-linked OPN fragments were obtained, one representing roughly the N-terminal half (OPN-O) and the other, the C-terminal half (OPN-P) of the protein (Fig 1A and 1B). Corresponding proteins of these fragments (OPN-O-V5, OPN-P-V5) were also expressed in insect cells (Fig 1C).

Ten hybridomas were produced from mice immunized against OPN-O or OPN-P. Of the clones that bound well to bacterial OPN-O, mAb 659 and mAb 446 are IgG antibodies which recognized linear epitopes (ELISA^+^, IFA^+^, and WB^+^), while mAb 163 is also an IgG but it recognized a conformational epitope (ELISA^+^, IFA^+^, WB^-^). All the OPN-P-specific antibodies (mAb 138, mAb 403, mAb 454, mAb 492) are IgMs which bound only to linear epitopes.

**Fig 2:**
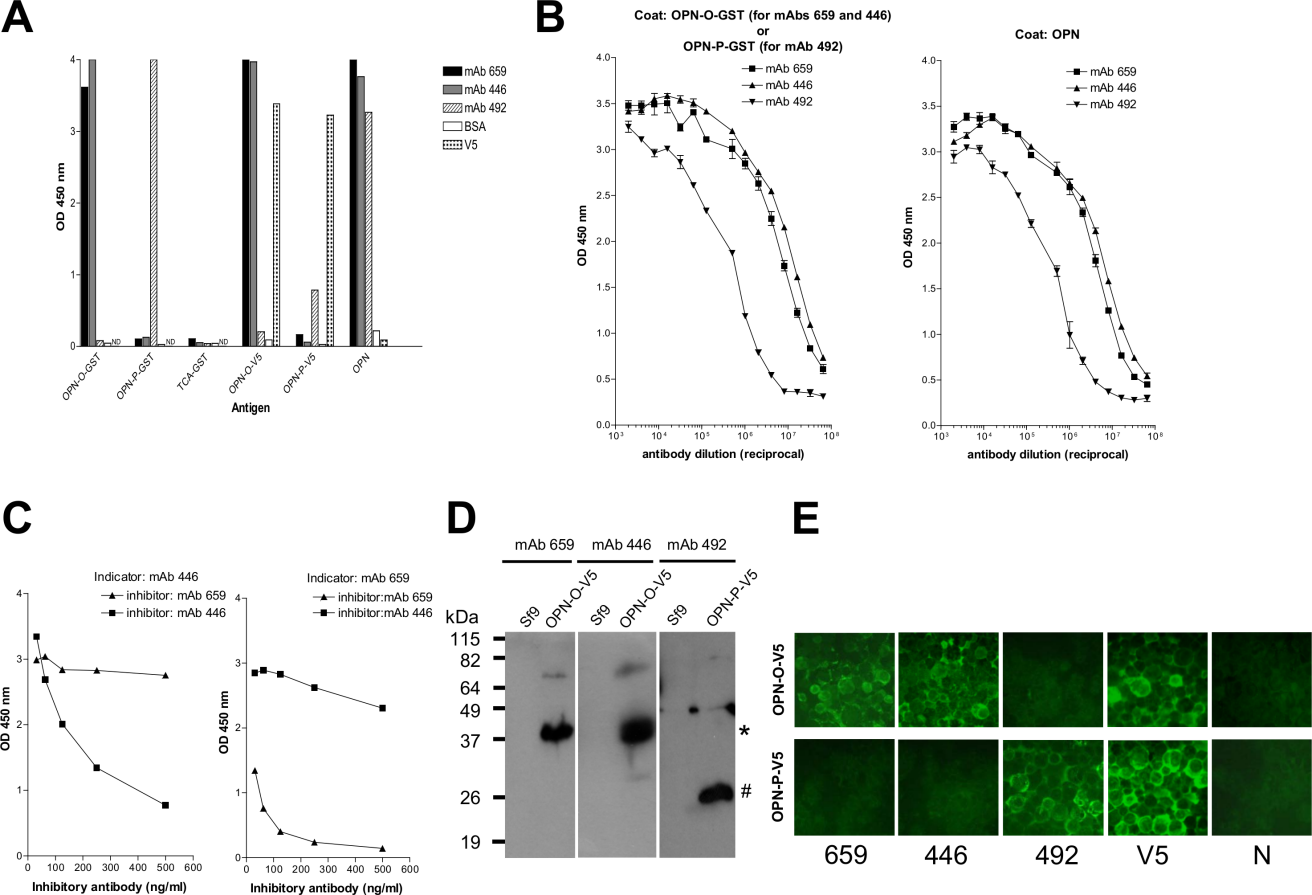
Characterization of the OPN-specific mouse mAbs. A) ELISA results showing binding specificity of the various mAbs (mAb 659, 446, and 492) to various OPN antigens (OPN-O-GST, OPN-P-GST, OPN-O-V5, OPN-P-V5, and full-length OPN), including TCA-GST (control negative containing human telomerase segment). Anti-V5 antibody (V5) and BSA are included as positive and negative control, respectively. B) ELISA results showing titration curves of individual mAbs (mAb 659, 446, and 492) against OPN-O-GST, OPN-P-GST or full-length OPN. C) Inhibition ELISA results showing mAb 659 and 446 do not cross-inhibit each other. Left panel shows the inability of mAb 659 to inhibit binding of biotin-labeled mAb 446 and the converse for right panel. D) Western blot results showing specific detection of the various insect cells-derived OPN antigens (OPN-O-V5, OPN-P-V5) by the various mAbs; 15% gel used; Sf9 is control insect-cell lysate. E) IFA results showing specific binding by various mAbs to insect cells expressing the appropriate OPN-O-V5 or OPN-P-V5 protein (magnification: 400 x). Anti-V5 antibody (V5) and normal mouse serum (N) used as positive and negative control, respectively.

mAb 659, mAb 446 and mAb 492 bound specifically in an ELISA to the recombinant OPN antigen derived from both bacteria and insect cells (Fig 2A and 2B); the binding was comparable to that for the full-length OPN obtained commercially (Fig 2B). mAb 659 and mAb 446 bound to different sites in OPN-O since they did not cross-inhibit each other (Fig 2C). With all three mAbs, specificity of binding was also demonstrated by WB analysis (Fig 2D) and immunofluorescence–staining of insect cells transfected with the respective OPN genes (Fig 2E).

### mAb 659 Recognizes the Thrombin-Sensitive Site in OPN

**Fig 3:**
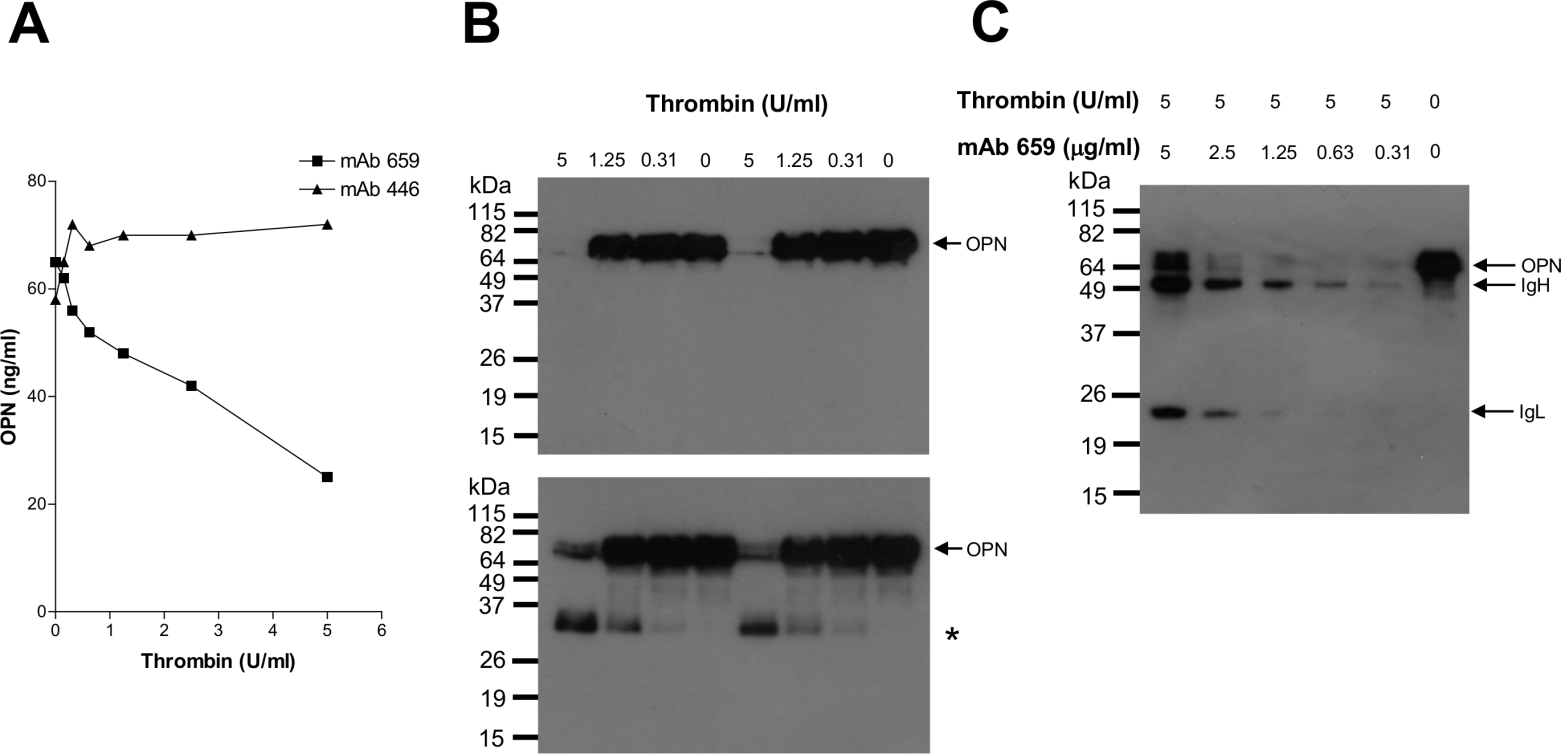
mAb 659 binds to the thrombin-cleavage site of OPN. A) ELISA results showing digestion of OPN by thrombin (0–5 U/ml), as evidenced by loss of antigenic activity determined using mAb 659 in an inhibition assay; such detection could not be made using mAb 446. B) WB results showing digestion of OPN by thrombin (0–5 U/ml) using mAb 659 (top panel) or mAb 446 (bottom panel) as probe. Digested fragment (*) revealed by mAb 446 but not mAb 659. Duplicate results shown (left and right panels). C) WB results of protection experiment showing the resistance of OPN to thrombin digestion when bound by mAb 659, using mAb 659 as probe.

We discovered, by chance, that mAb 659 recognized the evolutionary-conserved and unique thrombin-sensitive site (Arg^168^-Ser^169^) in OPN (see Fig 1A). First, it is known that digestion of OPN by thrombin produces two fragments of roughly equal size. Accordingly, we incubated intact OPN (obtained commercially) with increasing amounts of thrombin and monitored the result using mAb 659 in an ELISA to detect how much intact OPN remained: increasing degradation was observed with increasing enzyme (Fig 3A). In contrast, this effect was not observable when probed with mAb 446 (Fig 3A). Secondly, using mAb 659 as probe, full-length OPN could be observed as a 72 kDa band in Western blots; this disappeared completely when the protein was pre-treated with 5 U/ml thrombin (Fig 3B, upper panel), while smaller amounts of thrombin had little or no effect. When mAb 446 was used to probe the same blot where 5 U/mL thrombin had been used, there was also significant reduction in the amount of full-length OPN but for reasons unclear, this did not disappeared completely; more importantly, an additional antigen of 35 kDa appeared (Fig 3B, lower panel). Thirdly, mAb 659 was able to protect OPN from thrombin digestion in a dose-dependent fashion (Fig 3C).

### Inhibition ELISA Using mAb 659 or mAb 446 as Reagent Antibody Efficiently Detects Bacterial-Recombinant OPN, Insect-Cell-Recombinant OPN, NSO-Recombinant OPN and Native (HL60-Derived) OPN

**Fig 4:**
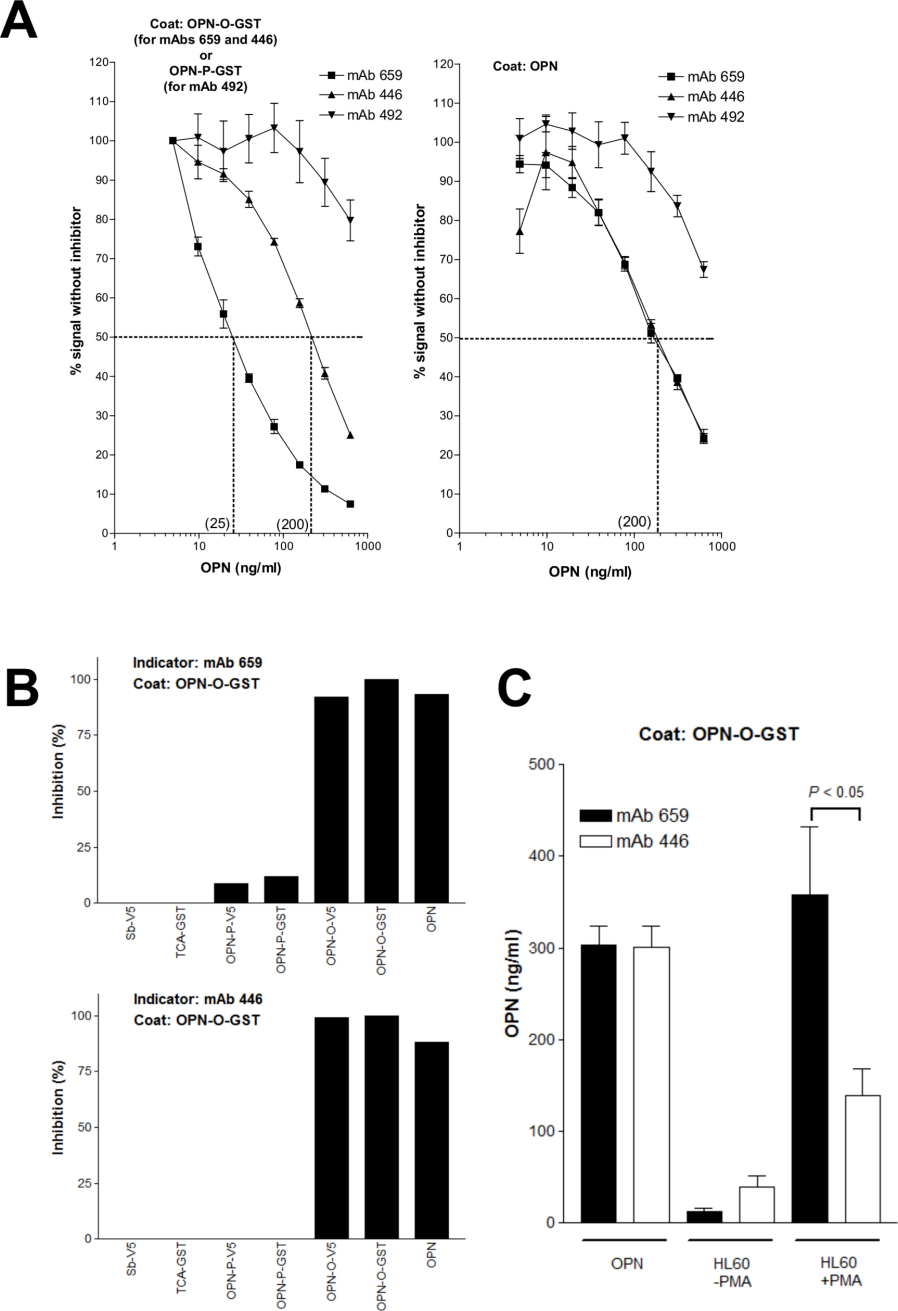
Detection of OPN in buffer solutions by various inhibition ELISAs. A) Dose-dependent inhibition of binding of various mAbs to target OPN (immobilized) by full-length OPN. Data represent mean +/- SD of three samples. B) Results showing specificity of the mAb 659 and mAb 446 inhibition ELISAs. OPN proteins (OPN, OPN-O-GST, OPN-O-V5, OPN-P-GST, and OPN-P-V5) and non-OPN proteins [TCA-GST and Sb-V5 (SARS-CoV spike protein)] were used as inhibitors. C) Comparison between the mAb 659 and mAb 446 inhibition ELISA on the detection of full-length OPN produced by HL60 cells [following stimulation or absence of stimulation with phorbol myristate acetate (PMA)]. NS0-derived recombinant human full-length OPN was used as positive control. Data represent mean +/- SD of three samples.

Inhibition ELISAs using mAb 659, mAb 446 or mAb 492 as the detecting antibody were developed to detect OPN by the ability of OPN to inhibit the binding of the mAb to the appropriate OPN substrate coated on the microplate (OPN-O-GST, OPN-P-GST, or full-length OPN). The concentration of detecting antibody used was pre-determined from the titration curves of these mAbs (data not shown), chosen at 70% maximal binding (Fig 2B). Full-length OPN obtained commercially or produced from mouse NS0 cells as histidine_6_-tagged proteins, was used as the standard. Assay sensitivity was determined as the concentration of full-length OPN (ng/mL) required to inhibit the maximal antibody binding by 50%. The sensitivities obtained for the different assays are shown in Fig 4A. Of particular note is the excellent detection by mAb 659 (25 ng/mL) when OPN-O-GST was used as substrate, compared to the poor detection by mAb 446 (200 ng/mL); both antibodies detected the commercially-obtained full-length OPN very poorly (200 ng/mL). Detection by mAb 492 was extremely poor with all OPN substrates.

Inhibition ELISAs were developed using either mAb 659 or mAb 446 as the reagent antibody and bacterially-produced OPN-O-GST as the substrate. Both assays efficiently and specifically detected the appropriate OPN produced from insect cells or mouse NS0 cells (Fig 4B). Interestingly, however, mAb 659 was 2.7 fold more sensitive than mAb 446 in detecting the OPN produced by HL60 cells (Fig 4C).

### Inhibition ELISA Using mAb 659 but Not mAb 446 Detects Significant Levels of Circulating OPN in Cervical Cancer Patients

**Fig 5:**
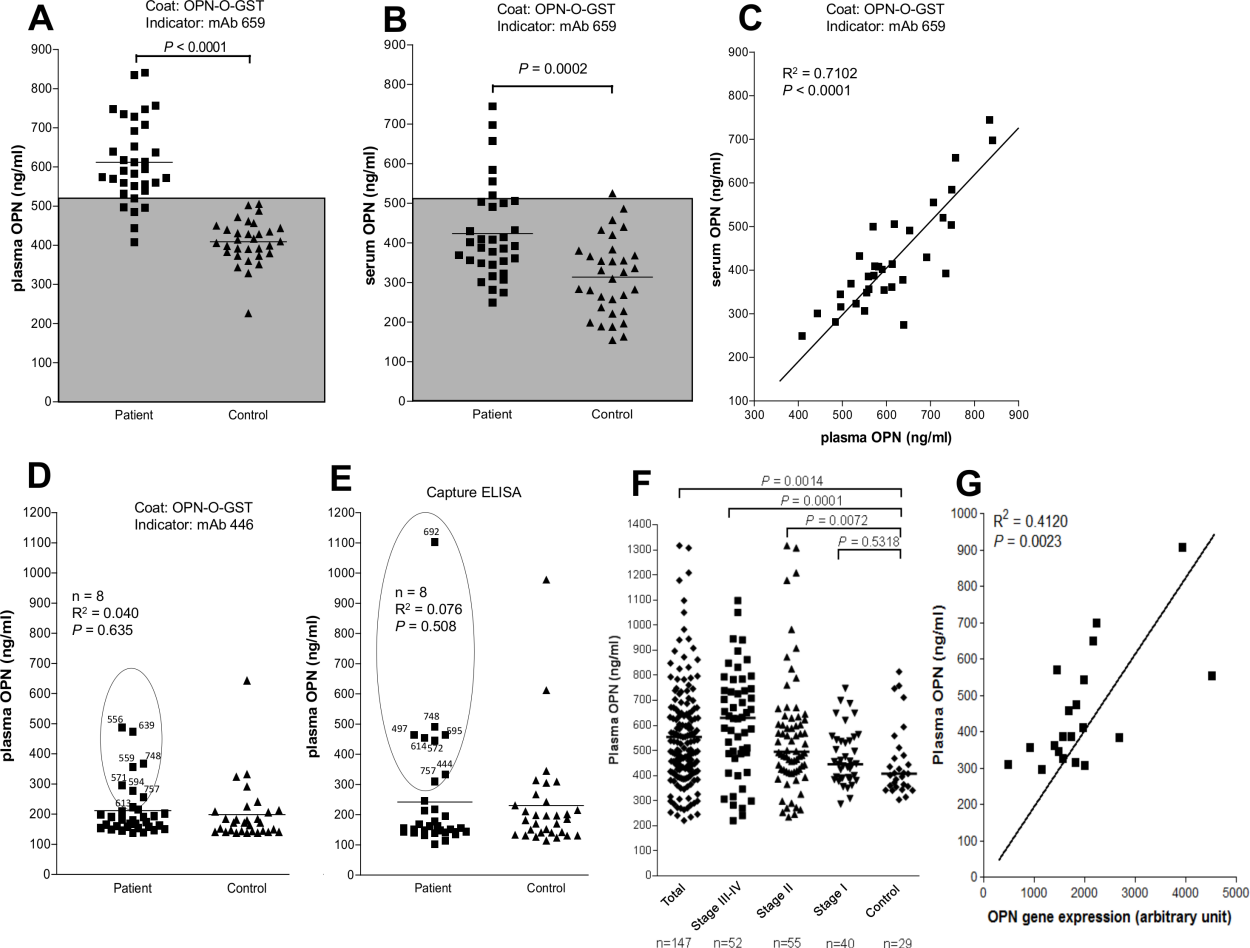
Comparison of various inhibition ELISAs and a commercial capture-ELISA in the detection of circulatory OPN from cervical cancer patients. A) Plasma OPN levels (ng/mL) detected by the mAb 659 ELISA from cervical cancer patients (n = 33) or normal subjects (n = 31). Shaded area denotes cut-off based on mean + 2 SD of OPN levels in normal subject. B) Same as in (A) except serum used instead of plasma. C) Regression analysis between plasma and serum OPN levels found in the cervical cancer patients. D) Same as in (A) except using the mAb 446 inhibition ELISA. The top eight responders are encircled with the OPN levels shown; these measurements were used in regression analysis against the OPN levels obtained from the mAb 659 inhibition ELISA–the results are indicated. E) Same as in (D) except using the commercial capture ELISA. F) Plasma OPN levels (ng/mL) detected by the mAb 659 inhibition ELISA from a new cohort of 147 cervical cancer patients and 29 healthy subjects; also shown is a breakdown of the patient group into the four stages of disease. In all cases, each point represents mean of duplicates. G) Regression analysis between plasma OPN levels and OPN gene expression levels determined from the tumor tissues of 20 cervical cancer patients.

We next used the mAb 659 OPN-O-GST inhibition ELISA to detect OPN from the plasma of 33 cervical cancer patients [26 squamous cell carcinoma, 5 adenocarcinoma, 2 cervical intraepithelial neoplasia (CIN) (grade III)]. We found highly significant (*P* < 0.0001) levels of OPN (mean +/- SD, 612 +/- 106 ng/mL) in these 33 patients compared to 31 healthy subjects (409 +/- 56 ng/mL) (Fig 5A). Based on sample size, this discrimination is highly significant (minimum no. of patients and control required for 99% confidence at 0.99 power is 30 and 12, respectively). The assay sensitivity was 82%, and the specificity, 100%. Similar results were found when the serum of these individuals was examined: the cancer patients (424 +/- 121 ng/mL) had significantly (*P* = 0.0002) higher levels of OPN than healthy subjects (314 +/- 98 ng/mL) (Fig 5B), the assay sensitivity and specificity being 18% and 97%, respectively. There is, in fact, good correlation (R^2^ = 0.71, *P* < 0.0001) between the plasma and serum OPN levels in the cervical cancer patients (Fig 5C), even though the serum levels were significantly lower than the corresponding plasma levels. However, based on sample size, the discrimination between patients and control is not robust (minimum no. of patients and control required for 85% confidence and 0.8 power is 42 and 28, respectively).

For comparison, when mAb 446 was used in the inhibition based on plasma, there was no discrimination between the cancer and healthy groups (Fig 5D). This was also the case using a commercially-available test kit, Human OPN Assay Kit (Immuno-Biological Laboratories Co. Ltd., Gunma, Japan), which employs a sandwich (capture) ELISA (Fig 5E). There was no correlation between the small number of patients found positive by this kit or the mAb 446 inhibition ELISA and the corresponding patients in the mAb 659 inhibition ELISA (Fig 5D and 5E).

We extended the mAb 659 inhibition ELISA study using more cervical cancer patients and plasma only, this time identifying the stage of the disease as well in order to make a more precise correlation between OPN level and disease. Thus, again, there was excellent discrimination (*P* = 0.0014) between the new cohort of 147 patients (560 +/- 211 ng/mL) and the 29 healthy subjects (mean 454 +/- 142 ng/mL) (Fig 5F). More interestingly, if the patients are subdivided according to the stage of the disease (Fig 5F), discrimination was seen in stage II (571 +/- 249 ng/mL; *P* = 0.0072) and, more pronouncedly, in stage III-IV (614 +/- 210 ng/mL; *P* = 0.0001), but not stage I (473 +/- 110 ng/mL; *P* = 0.5318). Tumor tissues were available from 20 patients (4 in stage I, 6 in stage II, 10 in stage I) in this cohort which were used to determine the OPN gene expression. As shown in Fig 5G, there was good concordance (R^2^ = 0.4120, *P* = 0.0023) between the plasma OPN results and the OPN gene expression results, thus validating the immunological detection.

### OPN Detected in Cancer Patients Is Fragmented but Contains the Intact Thrombin-Sensitive Site

**Fig 6:**
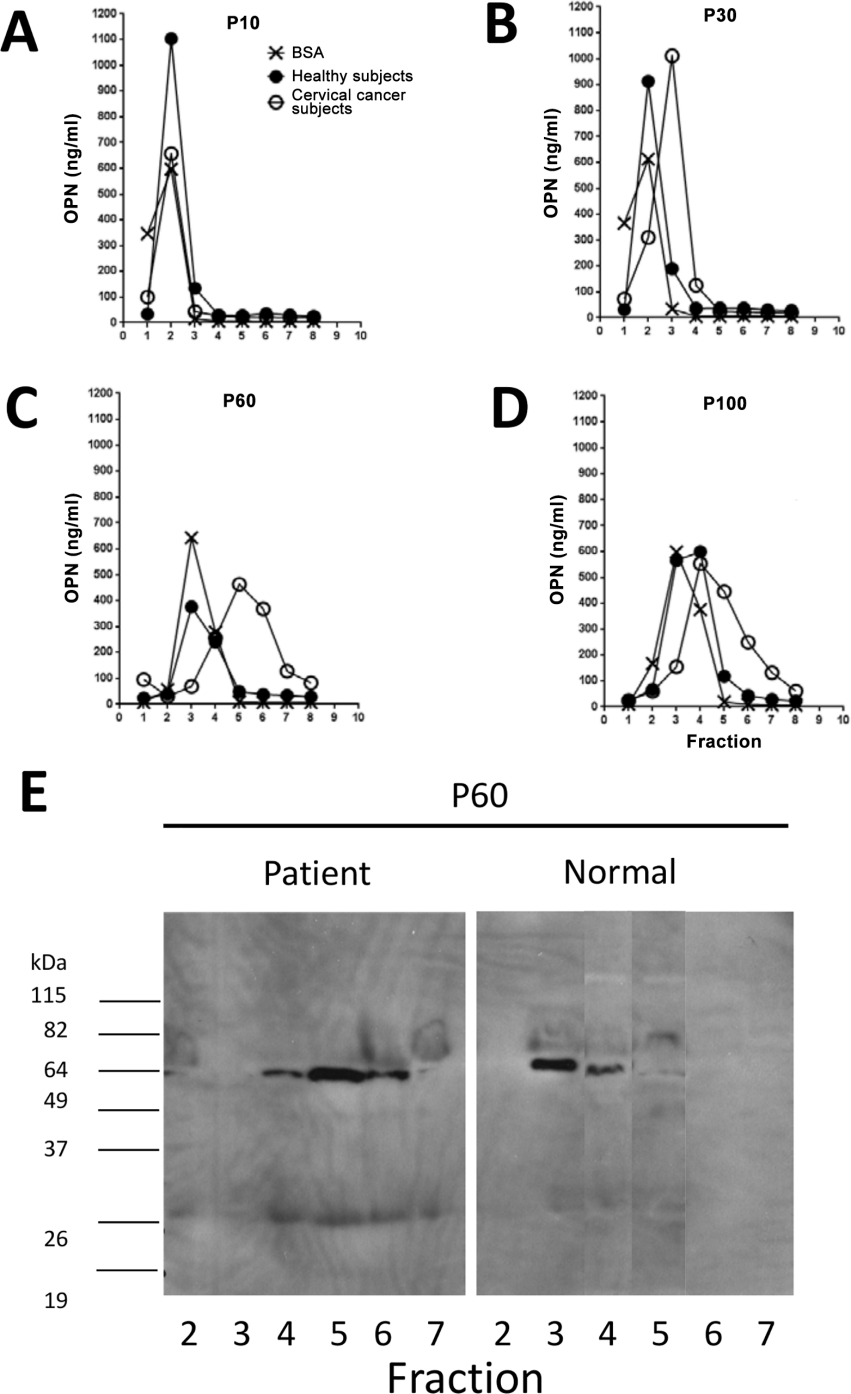
Gel fractionation of plasma OPN from cervical cancer patients and healthy subjects. Gel chromatography of pooled plasma from cervical cancer patients or healthy subjects using Bio-Gel of different retention limit: (A) P10 [20 kDa], (B) P30 [40 kDa], (C) P60 [60 kDa], and (D) P100 [100 kDa]. OPN antigenic content determined by mAb 659 inhibition ELISA; concentration of BSA (included as control) determined by BCA. (E) Western blot results showing the antigen detected in individual P60 gel fractions from patients or healthy people, probed by mAb 659.

The finding that the mAb 446 inhibition ELISA and the commercial OPN kit could not detect elevated OPN levels in the cancer patients suggested the possibility that the OPN present could be fragmented i.e. the target sites for the antibodies used in these assays could be missing, whereas, by virtue of the design of the mAb 659-based assay, the thrombin-sensitive site must be present. Thus, we fractionated the plasma of cancer patients by gel chromatography using a small but long, thin column made of Bio-Gel of different pore sizes (P10, P30, P60, P100). Serial 0.1 ml fractions were collected and assayed for OPN activity using the mAb 659 inhibition ELISA. Each column was pre-calibrated with blue dextran, DNP-lysine and BSA [a protein of similar molecular size (66.4 kDa) to intact OPN (around 70 kDa)]. As shown in Fig 6A, BSA was totally excluded in P10 (exclusion limit, 20 kDa) and P30 (40 kDa); in both cases, the bulk (60%) of the protein appeared in fraction 2 and the rest in fraction 1. In the P60 (60 kDa) and P100 (100 kDa) elution, however, BSA was not excluded but instead appeared mainly (58%) in fraction 3 and in smaller amounts in fraction 4, but none thereafter.

When the plasma pooled from four cervical cancer patients (selected on the basis of high OPN activity) was similarly fractionated, only in P10 was OPN totally excluded, with the bulk of the activity (82%) appearing in fraction 2 (Fig 6A). Significantly, in P30, the main OPN activity (67%) shifted to fraction 3. In the P60 and P100 elution, however, very little activity was recovered from this fraction (fraction 3); instead, there was a spread of activity from fraction 3 to fraction 8, peaking at fraction 4 (P100) or fraction 5 (P60). Collectively, the findings suggest that, in the cancer patients, OPN exists not as an intact protein but rather as fragments of between 40 kDa and 60 kDa in size.

Interestingly, when the plasma pooled from 4 healthy individuals was fractionated as above, the overall fractionation profiles obtained (Fig 6A) were slightly different from those of the cancer patients. Thus, total exclusion of OPN activity was observed with both P10 and P30, and the spread of activity in the included fractions of P60 and P100 was more limited (confined to fractions 3–5). Collectively, this suggests a protein size very similar to that of BSA (60–100 kDa).

We performed Western blotting of the P60 fractions obtained from both patients and control subjects (Fig 6B) using mAb 659 as probe. The results are consistent with those of the ELISA detection. Significantly, in the patients, no activity was detected from fractions 2 and 3, but fraction 4 revealed an antigen of about 64 kDa in size that was present in small amounts. Fraction 5 contained a marginally-smaller antigen (62 kDA) in significantly greater abundance, while the antigen found in modest amounts in fraction 6 was in turn marginally smaller in size (60 kDa). Fraction 7 had no activity.

In contrast, by Western blotting, in healthy subjects, the antigenic activity appeared earlier, in fraction 3 (close to the void volume of the column), which forms the bulk of the activity from the sample, while the molecular size of this antigen (roughly 68 kDa) is bigger than those of the fragments found in patients. A smaller antigen of about 60 kDa was found in smaller quantities from the subsequent fraction (fraction 4) while no activity was observed thereafter.

## Discussion

We sought to answer an important question in cancer diagnosis: is OPN present at diagnostic levels in the blood of cervical cancer patients? We found convincing evidence proving this. However, this was only true because we had used a very unique mAb in a highly-sensitive but not-so-common detection system. This mouse antibody (mAb 659) is highly specific for the unique thrombin-sensitive site in OPN, and OPN is efficiently detected from a patient by its ability to block the binding between this antibody and the target antigen. Thus, although healthy people were found to have nominal amounts of OPN in their blood in the assay, cancer patients showed significantly higher levels depending on several factors. This discrimination was found in two separate cohorts of cervical cancer patients. Plasma samples gave better results than serum in this assay, the reason being the fact that, as the blood clots to form serum, thrombin is activated which can digest the OPN in the sample. Presumably the conditions were not met for complete degradation of the protein. Validity of the plasma mAb 659 inhibition ELISA results is shown not only by the good correlation between these results and those of the corresponding serum samples, but also between these and the OPN gene expression results. Further validation is seen when the patients are re-grouped according to the stage of the disease: the OPN levels found by the ELISA increased with the severity of the disease. Thus, the most severe of these, stage III-IV, showed the highest level of antigen, while at the other extreme, the least severe, stage I, could not be distinguished from healthy individuals. Indeed, the ability to detect stage I patients possibly poses the greatest challenge to all of immunological assays.

The most important revelation of the study which sets it apart from past publications is the finding that OPN circulates in our patients not as an intact protein but rather, as fragments of about 60–64 kDa in size. These OPN fragments, which by inference must bear the intact thrombin-sensitive site, are probably truncated somewhere at the N-terminal end of the protein based on the observation that two immunoassays used in parallel with the mAb 659 inhibition ELISA had failed to detect similar OPN increases in the cancer patients: (1) An inhibition ELISA which uses mAb 446 as the detecting reagent known to bind to the N-terminal half of OPN (actual location unknown). (2) A commercially-available capture ELISA (Human OPN Detection Kit, IBL) which employed polyclonal antibodies directed at two sites in OPN separated by 138 amino-acids, one of which (^17^IPVKQADSGSSEEKQ^31^) is situated at the N-terminal end of the protein (Fig 1). Since this capture assay detects NS0-derived full-length OPN as efficiently as the mAb 659 inhibition ELISA, a likely reason for its failure is the absence of the N-terminal site in the patient’s OPN. The fact that this commercial kit was successfully used with other types of cancer previously [26] suggests that OPN might be cleaved differently (by different enzymes) in different types of cancer or in different individuals.

There are many OPN detection kits in the market but they all invariably use capture (sandwich) ELISA. They can be different, however, in the pair of antibodies utilized not only in terms of fine specificity but also whether these are polyclonal or monoclonal in nature; most polyclonal antibodies target a very small peptide-segment of the protein. Differences in the antibody pairs used can affect the type of OPN fragment detected and hence the vast discordances among test kits for the same set of plasma samples [32].

The inhibition ELISA we developed is the first of its kind for OPN detection. A distinct advantage of this format is the fact that the target site needs only be present in fragments or peptides as small as the site itself. It is instructive that at the start of our study, we had actually experimented with pairs of mAbs (e.g. biotinylated-mAb 659 and -mAb 446) in capture ELISAs to detect plasma OPN from patients, but none had succeeded. In general, inhibition assays are not common, but one which has proven efficacious in detecting typhoid fever is based on a rapid dual-particle system (‘TUBEX’) to detect a single specificity of antibody [36].

The OPN fragments were indirectly identified from pooled patient plasma by gel filtration. Although the results are preliminary and need to be verified more robustly in terms of sample size and methodology, they nevertheless merit some discussion. To compensate for the imprecision of the fractionation, the Bio-Gel columns were carefully calibrated using known markers and the results were based on a comparison of the plasma samples between the cancer and healthy groups run under identical conditions. Against this background, a notable difference in the P30 elution profiles between the two groups was indeed found: whereas in healthy people the bulk of the OPN antigenic activity appeared in the void fraction (fraction 2), in the cancer group, this appeared later (fraction 3)—suggesting an antigen smaller in size i.e. cleaved. Support for this is seen when the P60 or P100 elution profiles between these groups are compared. Thus, in the case of P60, whereas in healthy people the main antigenic activity was found in fraction 3, this appeared much later in fraction 5 in the cancer group, and there was a greater spread of activity. Western blot analysis performed on the P60 fractions using the same antibody probe (mAb 659) confirmed the ELISA findings. Thus, the main antigenic activity was found in fractions 3 and 5 from the healthy and cancer groups, respectively. More instructively, the antigen found in patients was significantly smaller (about 62 kDa) than that observed in healthy people (about 68 kDa). It seems likely the latter is the intact, full-length OPN even though it is slightly smaller than the intact OPN (72 kDa) produced by HL60 cells used early in our study–this difference could be due to post-translational modification (see later). Thus, the 62 kDa antigen found in patients is not intact but a major fragment. Interestingly, this fragment was also observed in healthy people as a minor component. In the patients too, other fragments (64 kDa in fraction 4 and 60 kDa in fraction 6) were also found in smaller quantities. It is possible that these three antigens are actually the same fragment but have varying degrees of post-translational modification. Such modifications can increase the molecular weight of the protein considerably. This is highly possible with OPN because although the protein is only roughly half as long (amino-acid) as BSA, it nevertheless has a very similar molecular weight. Indeed, OPN is known to be extensively phosphorylated because of which it is highly active biologically [37, 38]. There are indeed many potential sites in the protein both for phosphorylation (about 36 sites) and for glycosylation (5 for O-glycosylation, 2 for N-glycosylation) [39–41]. In addition, the protein also undergoes sulfation and transglutamination.

A possible candidate for the antigen found in the patients is the major fragment cleaved from whole OPN by caspase-8. This enzyme cleaves OPN at two sites, Asp135 and Asp 157 (Fig 1), yielding two major peptides of 180 and 160 amino-acid long, respectively, but both could yield molecular weights greater than 40 kDa [15] depending on the extent of post-translational modification. While these glycan or phosphate appendages mattered importantly to the linearized antigen in Western blotting by way of increasing the molecular weight, they do not seem to affect the molecular size of the free-form antigen in solution. Thus, in the P30 gel chromatography, the OPN antigen appeared more like a globular protein with a molecular weight of less than 30–40 kDa (i.e. a peptide with fewer than 270 amino-acids) than a 60 kDa antigen as deduced by Western blotting. The possibility that this antigen could be retarded in its passage through the gel due to interaction between the appended phosphate or carbohydrate groups and the Bio-Gel resin is belittled by the observation that the OPN antigen from healthy people seemed to be unaffected.

The surprising finding is the absence of intact OPN in our patients. The argument that this could be present but is bound at the thrombin-sensitive site by a co-factor such as syndecan-4 [42] or Factor H [43] and becomes masked as such, is ruled unlikely by the Western blot results. This is because any factor bound to OPN would have dissociated from it under the denaturing conditions used.

The question arises whether the OPN fragments found in our patients play a role in the cancer biology of the patient. On the one hand, this seems unlikely because the same fragment found in patients was also present in healthy people. Indeed, the greater abundance of this fragment in patients may simply reflect the general heightened activity of tumor cells–not only more OPN is produced, but also very quickly it becomes cleaved by the caspases and other enzymes in the tissue with the end result that vast amounts of OPN fragments are generated and released to the circulation. It is not clear, however, how intact OPN produced by other (non-cancerous) tissues becomes fragmented in our patients. On the other hand, there are numerous reports which described the potent biological activities of OPN fragments. This includes the OPN fragments generated by caspase-8, which bear the RGD domain (Fig 1) that was recently shown to promote tumor growth and metastasis [44]. Another OPN fragment which could remotely fit as the candidate antigen in our patients is the 32 kDa C-terminal peptide generated by MMP-3, MMP-7 or MMP-9 (see Fig 1) [45]; this, again, could be bigger in molecular weight if it becomes glycosylated or phosphorylated. The tumorigenic potential of this fragment is not known but recently, Takafuji et al. [17] found a small OPN fragment (residues 167–210) generated by MMP-9 which seemed able to induce tumor cell invasion via CD44 receptors in hepatocellular carcinoma [46].

Thus, the OPN fragments which circulate in our cancer patients and which bear both the RGD domain and an intact thrombin-sensitive site, may be more important than just a diagnostic (or prognostic) biomarker–they could very well determine the biology of the cancer.

## Abbreviations

OPN: osteopontin
mAb: monoclonal antibody
WB: Western blotting
ELISA: enzyme-linked immunosorbent assay
GST: glutathione S-transferase
SDS-PAGE: sodium dodecyl sulfate-polyacrylamide gel electrophoresis
HRP: horseradish peroxidase
FITC: fluorescein isothiocyanate
V5: amino acid residues ^95^GKPIPNPLLGLDST^108^ of RNA polymerase alpha subunit of simian virus 5
PMSF: phenylmethylsulphonyl fluoride
TMB: 3,3’,5,5’-tetramethylbenzidine
PMA: phorbol-12-myristate-13-acetate
